# The Hypouricemic Effect of *Balanophora laxiflora* Extracts and Derived Phytochemicals in Hyperuricemic Mice

**DOI:** 10.1155/2012/910152

**Published:** 2012-06-19

**Authors:** Shang-Tse Ho, Yu-Tang Tung, Chi-Chang Huang, Chao-Lin Kuo, Chi-Chen Lin, Suh-Ching Yang, Jyh-Horng Wu

**Affiliations:** ^1^Department of Forestry, National Chung Hsing University, Taichung 402, Taiwan; ^2^Department of Life Sciences, National Chung Hsing University, Taichung 402, Taiwan; ^3^Graduate Institute of Sports Science, National Taiwan Sport University, Taoyuan 333, Taiwan; ^4^School of Chinese Pharmaceutical Sciences and Chinese Medicine Resources, China Medical University, Taichung 404, Taiwan; ^5^Institute of Medical Technology, College of Life Science, National Chung Hsing University, Taichung 402, Taiwan; ^6^School of Nutrition and Health Sciences, Taipei Medical University, Taipei 110, Taiwan

## Abstract

The objective of this study is to evaluate the lowering of uric acid using *Balanophora laxiflora* extracts and derived phytochemicals on potassium-oxonate-(PO-) induced hyperuricemia in mice. The results revealed that ethyl acetate (EtOAc) fraction of *B. laxiflora* extracts exhibited strong xanthine-oxidase-(XOD-) inhibitory activity. In addition, among the 10 subfractions (EA1–10) derived from EtOAc fraction, subfraction 8 (EA8) exhibited the best XOD-inhibitory activity. Four specific phytochemicals, 1-*O*-(*E*)-caffeoyl-**β**-D-glucopyranose (**1**), 1-*O*-(*E*)-*p*-coumaroyl-**β**-D-glucopyranose (**2**), 1,3-di-*O*-galloyl-4,6-(*S*)-hexahydroxydiphenoyl-**β**-D-glucopyranose (**3**), and 1-*O*-(*E*)-caffeoyl-4,6-(*S*)-hexahydroxydiphenoyl-**β**-D-glucopyranose (**4**), were further isolated and identified from this subfraction. Compounds **3** and **4** exhibited the strongest XOD-inhibitory activity compared with other compounds, and both hydrolyzable tannins were determined to be noncompetitive inhibitors according to the Lineweaver-Burk plot. On the other hand, the *in vivo* hypouricemic effect in hyperuricemic mice was consistent with XOD-inhibitory activity, indicating that *B. laxiflora* extracts and derived phytochemicals could be potential candidates as new hypouricemic agents.

## 1. Introduction

Xanthine oxidase (XOD) is an important enzyme in humans that plays a crucial role in mediating purine metabolism. The major function of XOD is to convert hypoxanthine to xanthine and xanthine to uric acid [1, 2]. Many studies have demonstrated that excessive amounts of uric acid in the human body lead to the formation of urate crystals, which can cause diseases, such as inflammation, gouty arthritis, and tophaceous gout [3–6]. An epidemiological study has shown a high prevalence of gout in male and geriatric groups [7]. XOD inhibitors are generally used for the treatment of gout and hyperuricemia, which can hinder enzyme reactions involved in uric acid synthesis, reduce the formation of uric acid and relieve symptoms of the aforementioned diseases [8, 9].

Recently, scientists have attempted to find new and safe XOD inhibitors from a wide variety of traditional herbal plants [10–12]. *Balanophora laxiflora* Hemsl. (Balanophoraceae), which is traditionally used as a medicinal plant to cure cough, metrorrhagia, and hemorrhoids, is widely distributed in woodlands from mid to low altitudes in Taiwan. In our previous study, we determined that the extract of *B. laxiflora* exhibited good antioxidant activity [13]. Furthermore, the *Balanophora* species contain an abundant amount of hydrolyzable tannins and phenolic compounds [13–18]. Many studies have shown that some of these phenolic compounds have strong XOD-inhibitory activity and that the therapeutic effects of these compounds may be due to their antioxidant activity and enzyme inhibitory ability [2, 3, 8, 10]. However, there are no prior reports on lowering uric acid using *B. laxiflora* extracts. Thus, in this study, the lowering of uric acid by *B. laxiflora* extract and its derived phytochemicals was evaluated in an *in vitro* XOD-inhibitory assay and *in vivo* potassium-oxonate-(PO-) induced acute hyperuricemic mouse model, and these results provide scientific evidence of hypouricemic efficacy for the first time.

## 2. Materials and Methods

### 2.1. Chemicals

Xanthine oxidase, sodium pyrophosphate, xanthine, and allopurinol were all purchased from Sigma Chemical Co. (St. Louis, MO, USA). All other chemicals and solvents, that were used in this experiment, were of analytical grade.

### 2.2. Plant Material

*B. laxiflora* was collected in Taichung County, in the midlands of Taiwan. The plant species was identified by Dr. Chao-Lin Kuo, and a voucher specimen (voucher no. 4672) was deposited in the herbarium of the China Medical University, Taichung, Taiwan.

### 2.3. Extraction and Isolation

A dried sample from the male flower of *B. laxiflora* (5.0 kg) was chopped into small pieces and extracted with 40 L of methanol (MeOH) by soaking for one week at room temperature (25°C) twice. The methanolic extract was decanted, filtered under vacuum, concentrated in a rotary evaporator, and lyophilized. The yield of crude extract obtained was 8.0%. The resulting crude extract of *B. laxiflora* was fractionated successively with ethyl acetate (EtOAc), *n*-butanol (BuOH), and water to yield soluble fractions of EtOAc (4.1%), BuOH (2.2%), and water (1.0%). The EtOAc soluble fraction of *B. laxiflora* was loaded onto a chromatography column (Geduran Si-60, Merck, Darmstadt, Germany) and eluted with a gradient of EtOAc/n-hexane and MeOH/EtOAc solvent systems, and 10 subfractions (EA1–10) were collected. Potential XOD-inhibitory phytochemicals from the EA8 were separated and purified by semipreparative HPLC using a PU-2080 pump (Jasco, Japan) equipped with a MD-2010 multiwavelength detector (Jasco, Japan) and a 4 *μ*m Synergi Polar-RP column (250 × 10.0 mm i.d.; Phenomenex, Torrance, CA, USA). The mobile phase was 100% MeOH (solvent **A**) and ultrapure water (solvent **B**). The elution conditions were as follows: 0–30 min of 30–60% **A** to **B** (linear gradient) and 30–40 min of 60–100% **A** to **B** (linear gradient) at a flow rate of 4 mL/min. The structures of compounds **1**–**4** were identified by MS (Finnigan MAT-95S, Germany) and NMR (Varian Unity Inova-600, USA). All spectral data were consistent with the literature [19–22]. 

### 2.4. Determination of XOD-Inhibitory Activity

XOD-inhibitory activity was measured according to the method of Tung and Chang [5]. Briefly, 798 *μ*L of 0.1 units of xanthine oxidase in buffer (200 mM sodium pyrophosphate/HCl, pH 7.5) and 2 *μ*L of the test samples or compounds in DMSO were mixed at 37°C for 5 min. Subsequently, 200 *μ*L of 0.6 mM xanthine in ultrapure water was added to the mixture to initiate the reaction. The reaction mixture was incubated at room temperature, and the absorbance was measured every 1 min for 5 min at 295 nm using an ELISA reader (Thermo Scientific, USA). Allopurinol was used as a positive control. Three replicates were performed for each test sample. The percent inhibition ratio was calculated according to the following equation: % inhibition = [(rate of control reaction − rate of sample reaction)/rate of control reaction] × 100.

### 2.5. Lineweaver-Burk Plot

The mode of XOD inhibition by phytochemicals from *B. laxiflora* was determined by the Lineweaver-Burk plot. First, 20 *μ*L of 15 mM Na_2_EDTA in buffer (50 mM KH_2_PO_4_/KOH, pH 7.4), 30 *μ*L of 50, 60, 70, or 80 *μ*M xanthine in 50 mM KOH, 5 *μ*L of the test samples (0, 60, 80, and 100 *μ*M) in DMSO, and 195 *μ*L of buffer were mixed in 96-well microplates. The reaction was initiated by the addition of 50 *μ*L of xanthine oxidase in buffer (0.1 units/mL). The reaction mixture was incubated at 37°C, and the absorbance at 295 nm was determined every 5 s for 5 min using an ELISA reader. All data obtained from enzyme assays were plotted using Excel (Microsoft Office 2007, Microsoft, Taiwan).

### 2.6. Potassium-Oxonate-(PO-) Induced Hyperuricemia in Mice

Male ICR mice with body weights of about 30 g (6 weeks old) were purchased from the Laboratory Animal Center of the Medical College of National Taiwan University (Taipei, Taiwan). Mice were given a standard laboratory diet and distilled water ad libitum. In addition, they were kept on a 12 h light/dark cycle at 22 ± 2°C. This study was conducted according to the institutional guidelines and approved by the Institutional Animal Care and Utilization Committee of National Chung Hsing University, Taiwan. The lowering of uric acid by the EtOAc fraction and its phytochemicals on PO-induced hyperuricemic mice was carried out according to the method of Chien et al. [10] with slight modifications. Test animals were injected intraperitoneally (i.p.) with PBS containing 200 mg/kg of PO 1 h before the administration of test samples to increase their levels of serum uric acid. Mice were randomly assigned into the following 8 groups for different treatments: (1) vehicle group (*n* = 6); (2) PO group (*n* = 7); (3) PO + allopurinol group (PO + Allo group, *n* = 7); (4) PO + EtOAc fraction group (PO + EtOAc group, *n* = 7); (5) PO + 1-*O*-(*E*)-caffeoyl-*β*-d-glucopyranose group (PO + CFGP group, *n* = 7); (6) PO + 1-*O*-(*E*)-*p*-coumaroyl-*β*-d-glucopyranose group (PO + CMGP group, *n* = 7); (7) PO + 1,3-di-*O*-galloyl-4,6-(*S*)-hexahydroxydiphenoyl-*β*-d-glucopyranose group (PO + GHDGP group, *n* = 7); (8) PO + 1-*O*-(*E*)-caffeoyl-4,6-(*S*)-hexahydroxydiphenoyl-*β*-d-glucopyranose group (PO + CHDGP group, *n* = 7). For a comparative study, allopurinol, EtOAc fraction, CFGP, CMGP, GHDGP, and CHDGP dissolved in DMSO were delivered i.p. at 1 h after PO administration in the same dosage at a concentration of 10 mg/kg. Blood samples were collected by retroorbital bleeding 3 h after PO injection. Then, all mice were sacrificed, and their serum uric acid levels were determined using a commercial kit from Randox Laboratories (U.K.). 

### 2.7. Statistical Analyses

The results were expressed as the mean ± SD (*n* = 3) or mean ± SEM (*n* = 6 or 7). The significant difference was calculated by Scheffe's test; *P* values <0.05 were considered to be significant.

## 3. Results and Discussion

### 3.1. The XOD-Inhibitory Activity of *B. laxiflora* Crude Extract and Derived Soluble Fractions

**Figure 1:**
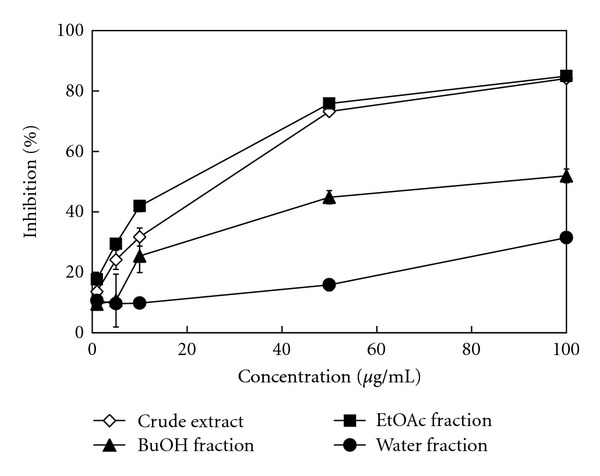
The XOD-inhibitory activity of *B. laxiflora* extracts and derived soluble fractions. The results represent the mean ± SD (*n* = 3).

The XOD-inhibitory activity of crude extract and its derived soluble fractions was dosedependent in Figure 1. In the presence of test samples at a concentration of 10 *μ*g/mL, the XOD-inhibitory activity decreased in the following order: EtOAc fraction (41.9%) > crude extract (31.7%) > BuOH fraction (25.4%) > water fraction (9.8%). The EtOAc fraction exhibited the strongest XOD-inhibitory activity. In addition, the IC_50_ values (the concentration required to inhibit uric acid formation by 50%) of crude extract, EtOAc fraction, BuOH fraction, and water fraction were 28.2, 14.2, 81.7, and >100 *μ*g/mL, respectively. Havlik et al. [2] reported that the leaf of *Thuja occidentalis* and fruit of *Prunus domestica* were historically used to treat gout in Europe. Furthermore, their 80% ethanolic extracts induced 43.5 and 3.4% XOD inhibition at a concentration of 200 *μ*g/mL. Additionally, the results reported by González et al. [11] showed that, at a concentration of 50 *μ*g/mL, the XOD-inhibitory activity of ethanolic extracts of *Crossopetalum lotabum* leaves, *Crossopetalum tonduzii* branches, and the whole plant from *Hyptis suaveolens* was 31, 42, and 52%, respectively. Kong et al. [23] reported that the XOD-inhibitory activity of water extracts from *Cinnamomum cassia* and *Morus alba* barks was 31 and 14%, respectively, at a concentration of 50 *μ*g/mL. Arimboor et al. [24] also reported that the methanolic extract from *Semecarpus anacardium*, an Indian traditional medicine used for the treatment of gout, rheumatoid arthritis, and inflammatory diseases, had potent XOD-inhibitory activity with an IC_50_ value of 253 *μ*g/mL. Comparisons of the aforementioned results indicated that there are abundant XOD-inhibitory phytochemicals present in the extracts of *B. laxiflora*, especially in the EtOAc fraction.

### 3.2. The Bioassay-Guided Isolation of the EtOAc Fraction from *B. laxiflora *Extracts

**Figure 2:**
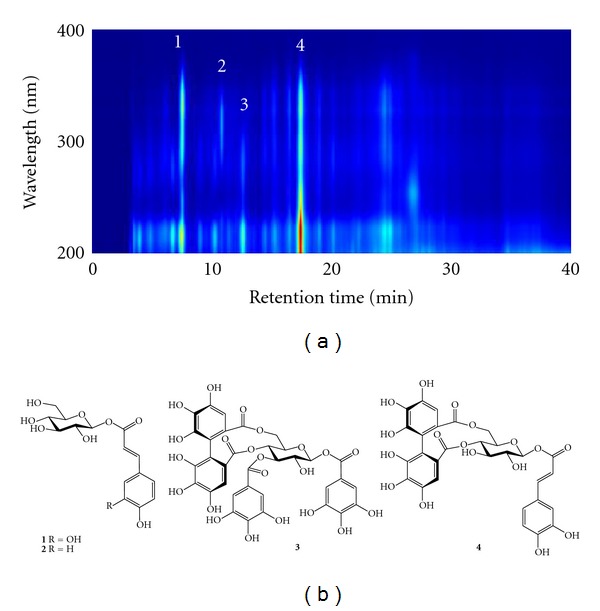
(a) HPLC-DAD chromatogram of EA8 subfraction from the EtOAc fraction of *B. laxiflora.* (b) The following phytochemicals were isolated from the EA8 subfraction: **1**, 1-*O*-(*E*)-caffeoyl-*β*-d-glucopyranose (CFGP); **2**, 1-*O*-(*E*)-*p*-coumaroyl-*β*-d-glucopyranose (CMGP); **3**, 1,3-di-*O*-galloyl-4,6-(*S*)-hexahydroxydiphenoyl-*β*-d-glucopyranose (GHDGP); **4**, 1-*O*-(*E*)-caffeoyl-4,6-(*S*)-hexahydroxydiphenoyl-*β*-d-glucopyranose (CHDGP).

Among *B. laxiflora* crude extracts and derived soluble fractions, the EtOAc fraction showed the best XOD-inhibitory activity. Using a bioassay-guided isolation, the constituents of the EtOAc fraction were further investigated in this study. The EtOAc soluble fraction was further divided into 10 subfractions by column chromatography. The elution solvent, collected weight, and XOD-inhibitory activity of these 10 subfractions are shown in Table 1. Of these subfractions, EA8 eluted with 100% of EtOAc and exhibited the strongest inhibitory activity (73.8%) against XOD at a concentration of 20 *μ*g/mL. Thus, the EA8 subfraction was isolated by semipreparative HPLC with diode-array detector (DAD).  Figure 2(a) shows the HPLC-DAD chromatogram of the EA8 subfraction from *B. laxiflora*. Four major constituents (Figure 2(b)) were purified and identified as 1-*O*-(*E*)-caffeoyl-*β*-d-glucopyranose (CFGP, 1), 1-*O*-(*E*)-*p*-coumaroyl-*β*-d-glucopyranose (CMGP, 2), 1,3-di-*O*-galloyl-4,6-(*S*)-hexahydroxydiphenoyl-*β*-d-glucopyranose (GHDGP, 3), and 1-*O*-(*E*)-caffeoyl-4,6-(*S*)-hexahydroxydiphenoyl-*β*-d-glucopyranose (CHDGP, 4) with yields of 0.47, 0.19, 0.31, and 0.50 mg per gram of dry *B. laxiflora*, respectively.

### 3.3. XOD-Inhibitory Activity of Phytochemicals from *B. laxiflora* Extracts

**Figure 3:**
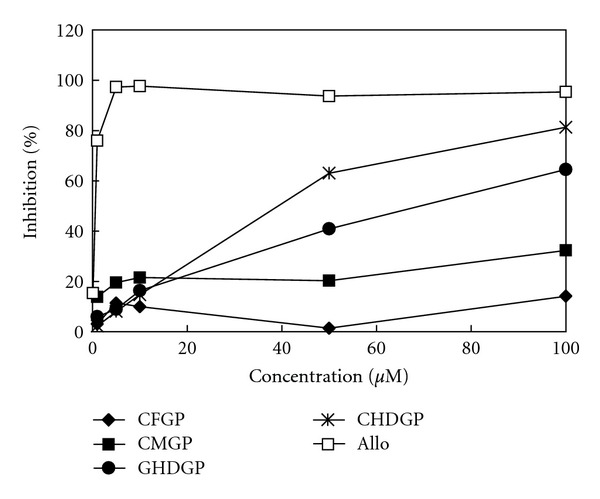
The XOD-inhibitory activity of phytochemicals from *B. laxiflora* extracts. Allo: allopurinol (positive control); CFGP: 1-*O*-(*E*)-caffeoyl-*β*-d-glucopyranose; CMGP: 1-*O*-(*E*)-*p*-coumaroyl-*β*-d-glucopyranose; GHDGP: 1,3-di-*O*-galloyl-4,6-(*S*)-hexahydroxydiphenoyl-*β*-d-glucopyranose; CHDGP: 1-*O*-(*E*)-caffeoyl-4,6-(*S*)-hexahydroxydiphenoyl-*β*-d-glucopyranose. The results represent the mean ± SD (*n* = 3).

**Figure 4:**
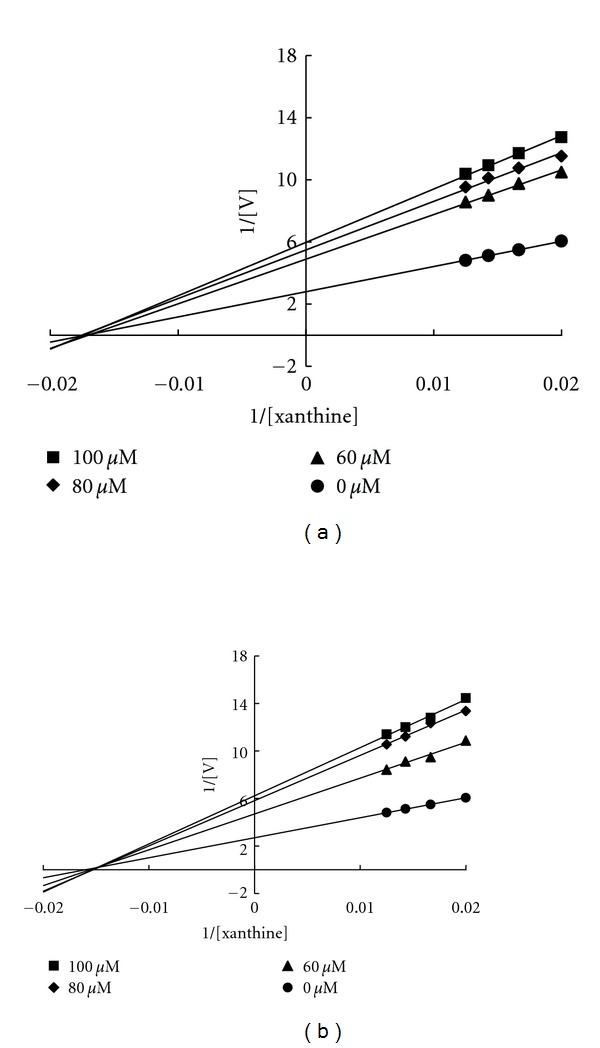
Kinetic assays of xanthine oxidase inhibition by GHDGP (a) and CHDGP (b). A Lineweaver-Burk double-reciprocal plot was constructed for the inhibition of xanthine oxidase by GHDGP and CHDGP. The plot represents 1/velocity versus 1/xanthine (*μ*M^−1^) in the presence or absence of phytochemicals in the reaction solution.

As shown in Figure 3, the XOD-inhibitory activity of phytochemicals from *B. laxiflora* extracts was compared with allopurinol, which is clinically used as an XOD inhibitor. Among the four phytochemicals, CHDGP (**4**) exhibited the best XOD-inhibitory activity (81.3%) at a concentration of 100 *μ*M, followed by GHGDP (64.5%) (**3**), CMGP (32.3%) (**2**), and CFGP (14.1%) (**1**). The IC_50_ values of compounds **1**–**4** and allopurinol were >100, >100, 70.9, 39.3, and 0.4 *μ*M, respectively. These results indicate that the XOD-inhibitory activity of phytocompounds **3** and **4** is much better than that of **1** and **2**. The chemical structures of **3** and **4** have a hexahydroxydiphenoyl moiety that might play an important role in enhancing their XOD-inhibitory activity. Unno et al. [25] reported that ellagic acid isolated from the aqueous extracts of the *Lagerstroemia speciosa* leaves exhibited excellent XOD-inhibitory activity with an IC_50_ value of 71.5 *μ*M. Lin et al. [26] reported that coumarin derivatives, including 4-methylesculetin (IC_50_ value of 75.8 *μ*M) and 4-hydroxycoumarins (IC_50_ value of 78.1 *μ*M), have strong XOD-inhibitory effects. Nguyen et al. [27] also reported that caffeic acid, eriodictyol, and 1,5-di-*O*-caffeoylquinic acid are potent XOD-inhibitors with IC_50_ values of 85.3, 43.8, and 64.4 *μ*M, respectively. In addition, some phenolic compounds, such as caffeic acid, ferulic acid, isoferulic acid, *p*-coumaric acid, and *p*-methoxycinnamic acid, were reported to have XOD-inhibitory activity with IC_50_ values of 65.6, 93.9, 143.2, 96.9, and 184.0 *μ*M, respectively [28]. These results reveal that hydrolyzable tannins GHDGP and CHDGP from *B. laxiflora* show excellent XOD-inhibitory activity. Furthermore, these tannins may be suitable for the treatment of XOD-related diseases. The Lineweaver-Burk plot was used to determine the kinetic mechanisms of inhibition by GHDGP and CHDGP. As shown in Figures 4(a) and 4(b), all straight lines in the Lineweaver-Burk plot intersect at the same point on the *x*-axis, which is characteristic of noncompetitive inhibition. Thus, these results indicate that the mode of XOD inhibition by both hydrolyzable tannins GHDGP and CHDGP is noncompetitive. In addition, the kinetic constants, K_*m*_ and K_*i*_, were determined from the Lineweaver-Burk and Dixon plots, respectively. Accordingly, the K_*m*_ for GHDGP and CHDGP were 57.6 ± 0.8 and 64.2 ± 1.4 *μ*M, and the K_*i*_ for GHDGP and CHDGP were 89.0 ± 2.0 and 70.8 ± 0.9 *μ*M, respectively.

### 3.4. The Hypouricemic Effect of *B. laxiflora* Extracts and Derived Phytochemicals in Hyperuricemic Mice

**Figure 5:**
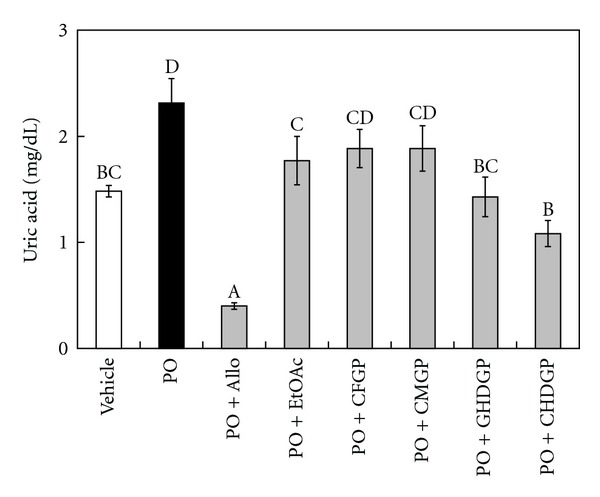
The lowering effect on uric acid by the EtOAc fraction and derived phytochemicals on PO-induced hyperuricemic mice. The results represent the mean ± SEM (*n* = 6 or 7). Bars marked with different letters are significantly different from each other (*P* < 0.05).

The hypouricemic effect of the EtOAc fraction and derived phytochemicals on PO-induced hyperuricemic mice is shown in Figure 5. In vehicle control mice, the serum uric acid level was 1.48 ± 0.13 mg/dL. In PO-induced mice (PO group), the serum uric acid level elevated to 2.31 ± 0.61 mg/dL after 3 h of PO injection. Thus, the serum uric acid level of PO group mice increased more than 1.5-fold as compared with the vehicle control. The administration of allopurinol (10 mg/kg) significantly reduced the serum uric acid level (0.40 ± 0.08 mg/dL) by 83% as compared with the PO group. At the same dosage (10 mg/kg), mice treated with the EtOAc fraction of *B. laxiflora* extracts (1.77 ± 0.60 mg/dL), GHDGP (1.43 ± 0.49 mg/dL), and CHDGP (1.08 ± 0.30 mg/dL) had uric acid levels that were significantly reduced by 23, 38 and 53%, respectively, relative to the PO group (*P* < 0.05). On the other hand, uric acid levels, between PO group and the animal treated with CFGP (1.89 ± 0.48 mg/dL) and CMGP (1.89 ± 0.57 mg/dL) at a concentration of 10 mg/kg, were not significantly different. Mo et al. [29] reported that at a dosage of 100 mg/kg quercetin, morin, myricetin, kaempferol, and icariin significantly reduced uric acid levels by 29, 31, 27, 35, and 13%, respectively. Wang et al. [30] reported that the administration of cinnamaldehyde (150 mg/kg) in hyperuricemic mice reduced 60% of the serum uric acid levels compared with the PO group. Comparisons of these results indicate that *B. laxiflora* extracts and their active components, GHDGP, and CHDGP, exhibit excellent hypouricemic effects. It is well known that XOD inhibitors are used to treat gout and hyperuricemia, which can hinder the enzymes involved in the synthesis of uric acid and reduce the formation of uric acid. Therefore, the hypouricemic effect of *B. laxiflora* extracts and derived phytochemicals may be due to their XOD-inhibitory activity. In addition, the *in vivo* lowering effect on uric acid was consistent with XOD-inhibitory activity, indicating that *B. laxiflora* extracts and derived phytochemicals possess potent hypouricemic effects and could be potential candidates for new hypouricemic agents.

## 4. Conclusions

In this study, the XOD-inhibitory activity and the hypouricemic effect of *B. laxiflora* extracts and derived phytochemicals were addressed for the first time. Four specific phytochemicals were isolated and identified from the EtOAc fraction of *B. laxiflora* extracts. The hydrolyzable tannins 1,3-di-*O*-galloyl-4,6-(*S*)-hexahydroxydiphenoyl-*β*-d-glucopyranose (GHDGP) and 1-*O*-(*E*)-caffeoyl-4,6-(*S*)-hexahydroxydiphenoyl-*β*-d-glucopyranose (CHDGP) exhibited potent XOD-inhibitory activity via noncompetitive inhibition. Furthermore, the hypouricemic effect of *B. laxiflora* extracts and derived phytochemicals was determined using a PO-induced hyperuricemic mouse model. Our results also revealed that the hydrolyzable tannins GHDGP and CHDGP significantly reduced serum uric acid levels. Accordingly, our results suggest that the bioactive phytochemicals from *B. laxiflora* extracts may represent a new type of hypouricemic agent and provide a potent hypouricemic effect for clinical use.

## Figures and Tables

**Table 1:** Mobile phase,yield, and XOD-inhibitory activity of 10 subfractions (20 *μ*g/mL) of the EtOAc fraction of *B. laxiflora*.

Subfractions	Mobile phase^a^ (v/v)	Yield (wt%)	Inhibition (%)^b^
EA1	5/95 (E/H)	4.2	3.7 ± 2.1^E^
EA2	10/90 (E/H)	1.6	16.6 ± 3.0^D^
EA3	25/75 (E/H)	2.3	19.5 ± 1.6^D^
EA4	25/75 (E/H)	0.5	24.6 ± 4.0^D^
EA5	25/75 (E/H)	0.5	42.7 ± 1.1^C^
EA6	50/50 (E/H)	12.7	54.7 ± 4.1^B^
EA7	75/25 (E/H)	28.9	68.5 ± 2.5^A^
EA8	100/0 (E/H)	18.9	73.8 ± 2.4^A^
EA9	10/90 (M/E)	16.9	68.4 ± 5.3^A^
EA10	30/70 (M/E)	5.7	51.3 ± 7.3^BC^

^a^E: ethyl acetate; H: *n*-hexane; M: methanol.

^b^The results represent the mean ± SD (*n* = 3). Different letters within a column indicate significant differences at *P* < 0.05.
